# Quantitative Expression of Key Cancer Markers in the AS-30D Hepatocarcinoma Model

**DOI:** 10.3389/fonc.2021.670292

**Published:** 2021-10-19

**Authors:** Marco A. Briones-Orta, Blanca Delgado-Coello, Roxana Gutiérrez-Vidal, Marcela Sosa-Garrocho, Marina Macías-Silva, Jaime Mas-Oliva

**Affiliations:** ^1^ Department of Infectious Disease, Imperial College London, London, United Kingdom; ^2^ Departamento de Bioquímica y Biología Estructural, Instituto de Fisiología Celular, Universidad Nacional Autónoma de México, Mexico City, Mexico; ^3^ Departamento de Biología Celular y Desarrollo, Instituto de Fisiología Celular, Universidad Nacional Autónoma de México, Mexico City, Mexico

**Keywords:** hepatocellular carcinoma, AS-30D cells, AS30D model, cancer markers, *Spp1*, osteopontin, epithelial-mesenchymal transition

## Abstract

Hepatocellular carcinoma is one of the cancers with the highest mortality rate worldwide. HCC is often diagnosed when the disease is already in an advanced stage, making the discovery and implementation of biomarkers for the disease a critical aim in cancer research. In this study, we aim to quantify the transcript levels of key signaling molecules relevant to different pathways known to participate in tumorigenesis, with special emphasis on those related to cancer hallmarks and epithelial-mesenchymal transition, using as a model the murine transplantable hepatocarcinoma AS-30D. Using qPCR to quantify the mRNA levels of genes involved in tumorigenesis, we found elevated levels for *Tgfb1* and *Spp1*, two master regulators of EMT. A mesenchymal signature profile for AS-30D cells is also supported by the overexpression of genes encoding for molecules known to be associated to aggressiveness and metastatic phenotypes such as *Foxm1*, *C-met*, and *Inppl1*. This study supports the use of the AS-30D cells as an efficient and cost-effective model to study gene expression changes in HCC, especially those associated with the EMT process.

## Introduction

Nowadays, hepatocellular carcinoma (HCC) is considered among the most frequent and aggressive cancer types, listed as the fourth cause of death worldwide when related to cancer (1). Considering the multifactorial character of cancer progression, its study becomes a challenging task that needs valuable models to study basic mechanisms related to proliferation, resistance to apoptosis, angiogenesis, and epithelial-mesenchymal transition (EMT), all hallmark features for the establishment and progression of cancer (2). In this study, employing the rat AS-30D hepatocarcinoma, we explore these hallmarks and phenotypical features of this model that are still unknown.

Established originally as a transplantable hepatoma model, the AS-30D cell, got its name because its ability to produce ascites fluid after 1 week of being inoculated, composed of cells of epithelial origin, morphologically similar to hepatocytes growing in clusters (3). Observations using electron microscopy show structures typical of epithelial cells, such as desmosomes and tight junctions, among others. Recent work shows that AS-30D cells injected directly into the liver of rats, develop solid tumors in defined sites of the liver parenchyma after 7 and up to 14 days of inoculation (4). Although neovascularization was confirmed after 7 days of inoculation, there is no evidence for the establishment of lung micrometastases even at day 14. As corresponding to highly malignant tumors, AS-30D cells show high glycolytic activity mediated by the type II hexokinase that becomes overexpressed after the promoter is demethylated (5, 6). Regarding the expression of cytokines regulating liver growth, AS-30D cells respond properly to TGF-β, and in comparison with normal hepatocytes, the Smad transcriptional corepressors Ski and SnoN, show larger half-lives (7). Another relevant signaling pathway present in the AS-30D model is PI3K-AKT-mTOR as mediator of the resistance of HCC to thermal ablative therapy (8), where the induction of EGFR signaling pathway has been demonstrated to be involved under heat stress conditions (9).

One of the main points of this work, is to explore the expression of osteopontin (OPN), also called secreted phosphoprotein 1 (*SPP1*, as the encoding gene), considered an extracellular matrix glycoprotein abundant in bones and teeth. In the last two decades, OPN function has been revisited, and nowadays, it is known to be a versatile molecule regulating several cellular processes such as migration, proliferation, apoptosis, and immune responses, among others (10, 11). According to this wide range of functions, OPN is expressed as several isoforms generated by alternative splicing. The protein also might go through several posttranslational modifications or can be processed by proteolytic cleavage into functional fragments.

OPN is overexpressed in several pathological processes affecting the liver, such as fibrosis, nonalcoholic or alcoholic fatty liver disease, cirrhosis, and cancer (12–14). It has been shown that liver samples from patients suffering these diseases, *Spp1* transcripts are enriched (15–18). The neutralization of OPN through antibodies or aptamers as a resource to successfully treat liver fibrosis has been shown (15). Importantly, OPN has been proposed as a prognosis molecule and biomarker when associated to HCC in both, the mouse and the human (19–22). Moreover, specific splice variants can differentially influence the progression and prognosis of the disease (23). During the early stages of cancer establishment where local inflammatory processes occur, OPN interacts with receptors such as CD44 and integrins, stimulating cell adhesion and migration, and therefore contributing to metastasis.

During our study, in an attempt to better understand the development of HCC, we used the hepatoma model AS-30D to analyze the expression pattern of *Spp1* transcripts and other genes critical in cancer progression and metastasis. These genes cover fundamental hallmarks in cancer as EMT, angiogenesis, cell proliferation, and extracellular protein matrix processing. While EMT involves the major cell trans-differentiation critical for cancer cell migration and metastasis (23), under normal conditions, EMT is essential during embryogenesis. Nevertheless, during metastatic process EMT is reactivated allowing cells to inhibit the normal cell-cell adhesion, and therefore becoming mobile and invasive cells (24, 25). Moreover, since it is well known that calcium homeostasis is altered in cancer cells (26), and a series of molecules involved with EMT such as cadherins and OPN, present a calcium-dependent activation, we also investigated if changes in genes codifying for the plasma membrane calcium ATPases (PMCA) named *Atp2b1-Atp2b4*, that in normal conditions maintain intracellular Ca^2+^ at the nanomolar range, could present a correlation with possible changes on *Spp1* expression. Supported on a series of studies carried out by our research group over the years (26–29), we hold the possibility that this correlation might be of key importance in the establishment of HCC and further progression of the disease.

## Materials and Methods

### Oncomine Database

The Oncomine database was used to explore the expression of osteopontin (*SPP1*) in human samples derived from normal liver and HCC (https://www.oncomine.org) (30). For the analysis, we used two datasets: Mas et al. (31), which includes 19 normal and 38 HCC samples, and Chen et al. (32), which includes 75 normal and 105 HCC samples. We also used Oncomine to compare the expression levels in normal and HCC samples of the genes included in this work: *VEGFA*, *ANGPT1*, *FOXM1*, *MET*, *INPPL1*, *EGRF*, *TGFB1*, *MMP2*, *CDH2*, *ACTA2*, *CD44*, *ITGA3*, *ITGA5*, *ATP2B1*, *ATP2B2*, *ATP2B3*, and *ATP2B4.* We used the dataset from Chen et al. (32), except for *ITGA5*, which is not included in this microarray; then, for *ITGA5*, we presented the data from Mas et al. (31).

### Reagents

All salts and buffers employed were from Sigma-Aldrich (St. Louis, MO, USA). The Trizol reagent was from Thermo Fisher Scientific (Waltham, MA, USA). The iScript cDNA synthesis kit was purchased from Bio-Rad (Hercules, CA, USA) and PowerUp Sybr Green Master Mix 2X from Applied Biosystems (Foster City, CA, USA). Several primers were used as reported previously (33–35) or designed using the platform of the Universal Probe Library from Roche (Pleasanton, CA, USA) (**Table 1**).

**Table 1:** Primer sequences used for qPCR experiments.

Gene	Forward (5’→3’)	Reverse (5’→3’)
*Rpl13*	AAAGCCTGCCAGAATTTCTCT	GGGCTCCAAGGACTCTCTG
*Vegfα* (33)	CAGCTATTGCCGTCCAATGA	CCAGGGCTTCATCATTGCA
*Angpt1*	ATGCGCCCTTATGCTAACAG	TTTAGATTGGAAGGGCCACA
*FoxM1*	CGAGGACCACTTCCCTTATTT	AGCTTGTGGTAGGCCTGGT
*Met (c-Met)*	CAGCGGTACATGGACTCAAG	TGCACTAGTGGGGAAAACCT
*Inppl1*	CGCACTCTGCGTCCTGTA	AGGTCTGCACAGCCAGGA
*Egfr*	TGCACCATCGACGTCTACAT	AACTTTGGGCGGCTATCAG
*Tgfb1*	GCAACACGTAGAACTCTACCAGAA	CAGCCACTCAGGCGTATCA
*Spp1* (34)	CCCATCTCAGAAGCAGAATCTCCTA	ATCATCCATATCATCCATGTGGTCA
*Mmp2*	CACCACCGAGGATTATGACC	CACCCACAGTGGACATAGCA
*Cdh2*	CCATCATCGCGATACTTCTG	CCATACCACGAACATGAGGA
*Acta2*	CCAGTCGCCATCAGGAAC	TGTGCTGTCTTCCTCTTCACA
*Cd44*	CTACTGGAGACCGGGATGAC	GGCAGAAACCCATGGAGTAG
*Itgβ3*	TGCTAAATTTGAGGAAGAACGA	CTTTATACAATGGGTTGTTTGCTG
*Itga5* (35)	GCACCATTCAATTTGACAGC	TTGTACTCCACAGGTTCCTCAC
*Atp2b1*	GCAATTGAAAATCGCAACAA	TTCAGAGGCTGCATCTCCAT
*Atp2b2*	CGGAGTGTGGACTAACAGCA	CTAGGGACTGCGTGACCAAG
*Atp2b3*	CAATCTTTCTACCCCTACTCACATC	GATGCAACTCTTGGATTCTGG
*Atp2b4*	CTCTGAAAATCGCAACAAAGC	AGTGGCTGGATTTCCAAGG

Rpl13, ribosomal protein L13; Vegfα, Vascular Endothelial Growth Factor α; Angpt1, Angiopoietin 1; FoxM1, Forkhead box M1; C-met, MET Proto-Oncogene, Receptor Tyrosine Kinase; Inppl1, Inositol Polyphosphate Phosphatase Like-1; Egfr, Epidermal Growth Factor Receptor; Tgfb1, transforming growth factor-β1; Spp1, Secreted Phosphoprotein 1 (OPN); Mmp2, metalloprotease 2; Cdh2, N-cadherin, Acta2, Actin, Alpha 2, Smooth Muscle, Aorta; Cd44, CD44 receptor; Itgb3, Integrin Subunit Beta 3; Itga5, Integrin Subunit Alpha 5; Atp2b1-Atp2b4, PMCA1-PMCA4 isoforms.

### Experimental Model

Experimental animals were handled according to the Mexican Official Norm for Laboratory Animals (NOM-062-ZOO-1999) and the procedures approved by the Animal Care and Use Committee of our Institute (Protocol JMO119(89)-17). To expand the transplantable hepatocarcinoma, a vial containing ~3 × 10^6^ AS-30D cells/ml preserved in Dulbecco’s modified medium plus 10% dimethyl sulfoxide was inoculated intraperitoneally to male Wistar rats (*n* = 4) weighing 200 g. After 1 week, rats developed ascites that was collected as described before (27). In order to eliminate erythrocytes, the ascitic fluid was washed several times with a buffer containing 150 mM NaCl, 5 mM KCl, and 20 mM Tris-HCl (pH 7.4) by centrifugation at 1,000×g for 10 min at 4°C. Clean pellets were resuspended in PBS or in Trizol, then frozen in liquid nitrogen and stored at −70°C until use. Fresh hepatocytes were isolated from male Wistar rats (*n* = 3) weighing 250 g as previously described (28). Briefly, rats were anesthetized intramuscularly with ketamine and xylazine (40–80 and 5–10 mg/kg, respectively). After perfusion of the liver with Krebs-Ringer solution containing 0.05% collagenase (Worthington, Lakewood, NJ, USA), the obtained cell suspension was filtered and centrifuged at 500 rpm during 2 min. Hepatocytes were recovered from the pellet, washed with Krebs-Ringer solution and snap frozen in liquid nitrogen and stored at −70°C.

### Quantitative PCR

Total RNA was obtained with Trizol, following manufacturer’s instructions, and quality of RNA samples was verified in 1.5% agarose gels. In order to synthesize cDNA, 1 μg of RNA was used for reverse transcription and reactions set using iScript RT Supermix as recommended by the provider. cDNA obtained from different tissues or cells was used for qPCR together with the PowerUp Sybr Green Master Mix 2X, according to manufacturer’s instructions. qPCR reactions and dissociation analysis were performed in an ABI PRISM 7000 Sequence Detection equipment from Applied Biosystems according to the standard cycling program suggested by the manufacturer, comprising a step for UDG inactivation for 2 min at 50°C, a dual-lock DNA polymerase step for 2 min at 95°C, and 40 cycles including two steps for 15 s at 95°C and for 1 min at 60°C. A preliminary validation was performed, including hepatic C9 cells and fresh hepatocytes; hepatocytes were determined appropriate as reference of normal expression of the analyzed transcripts. The housekeeping gene *Rpl13* (60S ribosomal protein L13) was used as reference and qPCR data were analyzed with the 2^−ΔΔCt^ method.

### Statistical Analysis

All samples were measured in triplicates; mean values, standard deviations (SD), and *t*-test comparison were calculated using the Graphpad Prism 8 software (San Diego, CA, USA). Statistically significant differences were those with *p-values ≤0.05; **p ≤ 0.01; ***p ≤ 0.001; and ****p ≤ 0.0001.

### Western Blot Assays and Cell Treatments

Fresh hepatocytes and AS-30D cells were lysed with RIPA buffer plus a cocktail of protease inhibitors (cOmplet protease Inhibitors, Roche, Basel, CHE) and phosphatase inhibitors (1 mM NaF, 1 mM NaVO3); 100 μg of total protein extracts were run in SDS-PAGE as described before (36). Antibodies used were the following ones: vimentin 5741, pSmad2 3108 (Cell Signaling Technology, Danvers, MA, USA), osteopontin AF808 (R&D Systems, Minneapolis, MN, USA), PMCA 1-4 sc-20028 (Santa Cruz Biotechnology, Dallas, TX, USA), and β-actin A2228 (Sigma-Aldrich, St. Louis MO, USA). Recombinant Human TGF-β was obtained from PeproTech (Rocky Hill, NJ, USA). AS-30D cells were cultured in DMEM with 10% FCS; cells were incubated with 2 ng/ml of TGF-β for 24 or 48 h.

## Results

### Spp1 Expression Analysis in Human HCC

**Figure 1:**
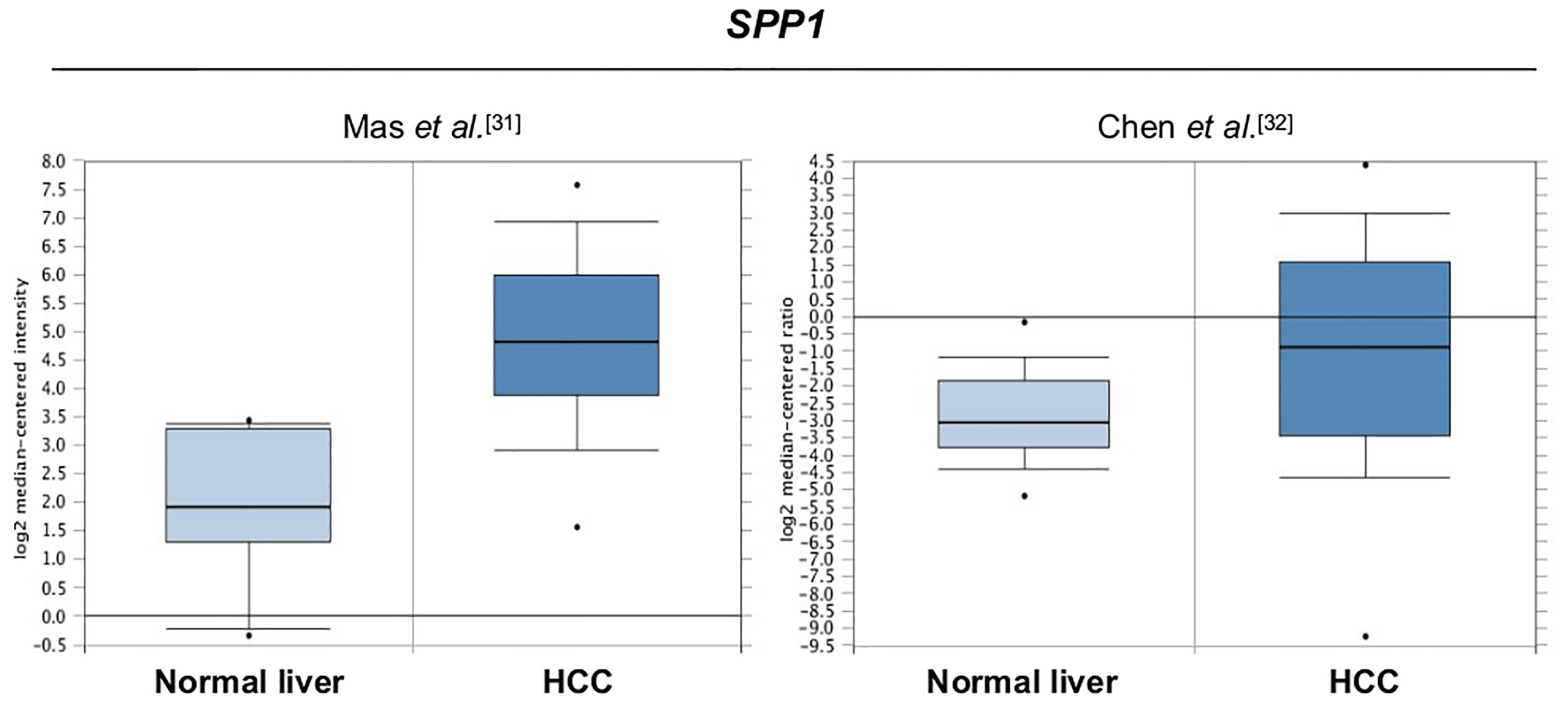
Comparative expression of *SPP1* in human HCC samples compared with normal liver. *SPP1* expression is 7.5-fold higher in HCC than in normal liver. Box plot obtained from Oncomine database (30).

First, to obtain information related to the latest findings on the relevance of *SPP1* in HCC, we compared the expression of this gene in normal liver and HCC samples using the database Oncomine (30). The level of *SPP1* expression in HCC samples was consistently higher in HCC compared with normal liver; specifically, 7.5 times higher in the dataset from Mas et al. (31) and 4.0 times higher in the dataset from Chen et al. (32) (**Figure 1**). To facilitate a comparison between human HCC and the hepatoma AS-30D, we obtained gene expression profile of all the genes analyzed in this study, using Oncomine with the dataset of Chen et al. (32) (**Supplementary Figure S1**).

### Gene Expression Analysis Performed in AS-30D Cells

Since the characterization of *Spp1* is still not available in HCC developed in the rat, we proceeded to quantify *Spp1*, and other genes related to HCC progression using the AS-30D cells as a model of HCC. cDNA from AS-30D hepatoma cells obtained from different specimens was prepared and amplified by qPCR in parallel with samples obtained from fresh rat hepatocytes, used as a basal expression control. For clarity purposes, the analyzed genes were grouped according to the specific physiological processes where they participate.

### Angiogenesis Markers

**Figure 2:**
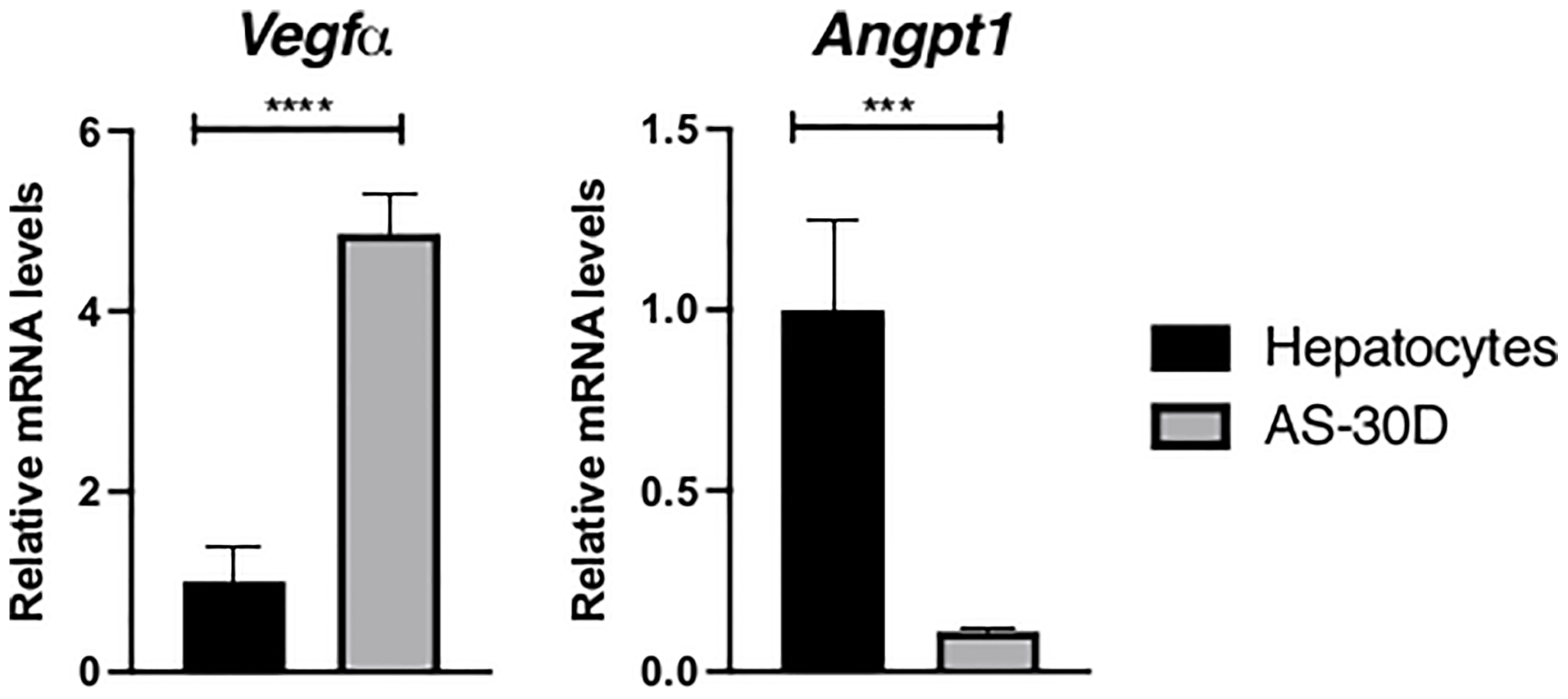
Expression of genes involved in angiogenesis in rat hepatocytes and AS-30D cells. Rat hepatocytes were freshly isolated, and AS-30D samples were obtained from cells from the ascitic fluid of experimental rats. Data represented as mean ± SD. ***p ≤ 0.001; and ****p ≤ 0.0001.

As part of this group of genes, *Vegfα* and *Angpt1* transcripts were studied finding a contrasting pattern between them (**Figure 2**). *Vegfα* mRNA showed a consistent higher expression in AS-30D samples in comparison with normal fresh hepatocytes, while *Angpt1* mRNA was highly expressed in normal hepatocytes, and lightly expressed in hepatoma cells.

### Cell Cycle and Metabolism Markers

**Figure 3:**
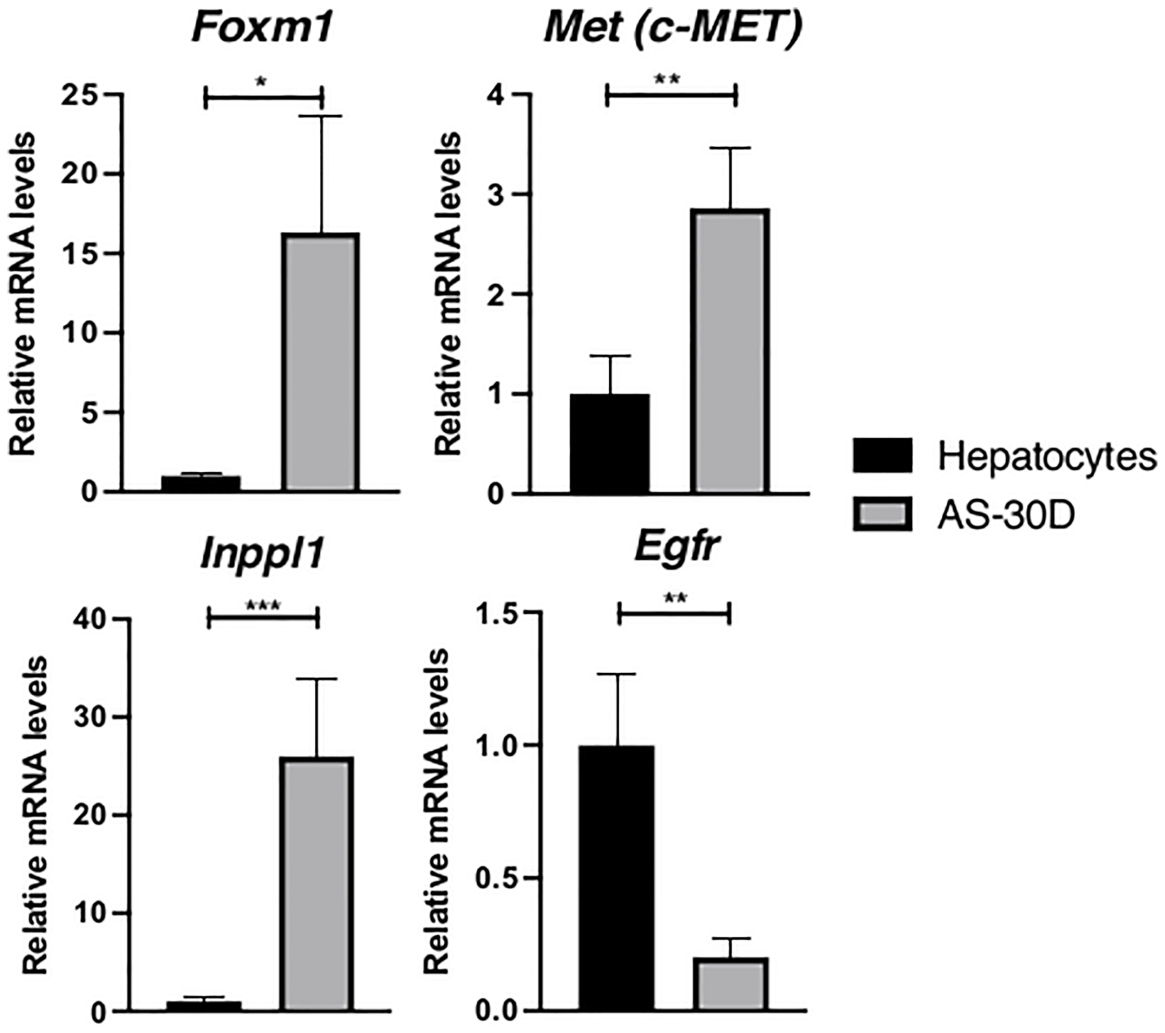
Expression of several genes related to metabolic processes or regulation of cell cycle in rat hepatocytes and AS-30D cells. Rat hepatocytes were freshly isolated, and AS-30D samples were obtained from cells isolated the ascitic fluid of experimental rats. Data represented as mean ± SD. *p-values ≤0.05; **p ≤0.01; ***p ≤ 0.001.

In this group of genes, we found a higher expression of *FoxM1*, *C-met*, and *Inppl1* (**Figure 3**). *FoxM1* gene encodes a protein corresponding to an important transcription factor for cell cycle progression and therefore, cancer (37). *C-met* that is overexpressed in cancer, encodes for the hepatocyte-growth factor receptor, a major regulator during embryogenesis and wound healing. *Inppl1* encodes for a phosphatase negatively regulating P13K pathways through the hydrolysis of the 5-phosphate of phosphatidylinositol-3,4,5-trisphosphate to convert it in phosphatidylinositol-4,5-diphosphate. This pathway is indicative of a high metabolic activity compatible with an aggressive type of cancer (38, 39). On the other hand, *Egfr*, that encodes for the epidermal growth factor receptor, is clearly underexpressed in hepatoma cells with respect to normal hepatocytes.

### EMT Markers

**Figure 4:**
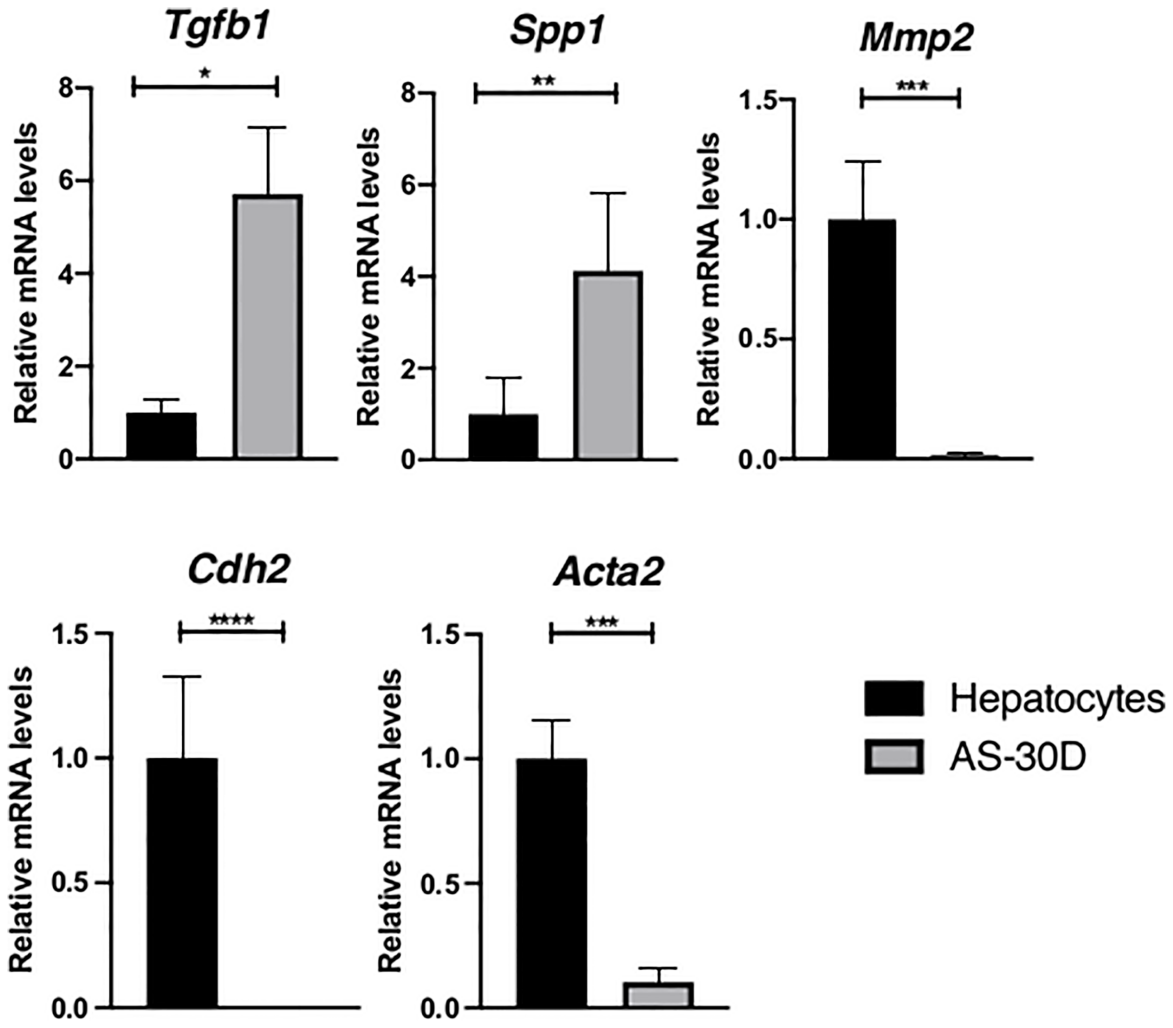
Expression of genes encoding EMT markers in rat hepatocytes and AS-30D cells. Rat hepatocytes were freshly isolated, and AS-30D samples were obtained from cells from the ascitic fluid of experimental rats. Data represented as mean ± SD. *p-values ≤0.05; **p ≤0.01; ***p ≤ 0.001; and ****p ≤ 0.0001.

Based on the fact that TGF-β is recognized as a major inductor of EMT (40), we explored the expression of this cytokine in our model. Interestingly, mainly *Tgfb1* and *Spp1* mRNA are highly overexpressed in the hepatoma cells with respect to the levels observed in normal fresh hepatocytes (**Figure 4**). In an opposite manner, mRNA levels of some EMT markers such as *Mmp2*, *Cdh2*, and *Acta2* are virtually absent in the ascites tumor cells.

### Extracellular Matrix and Cell Adhesion Markers

**Figure 5:**
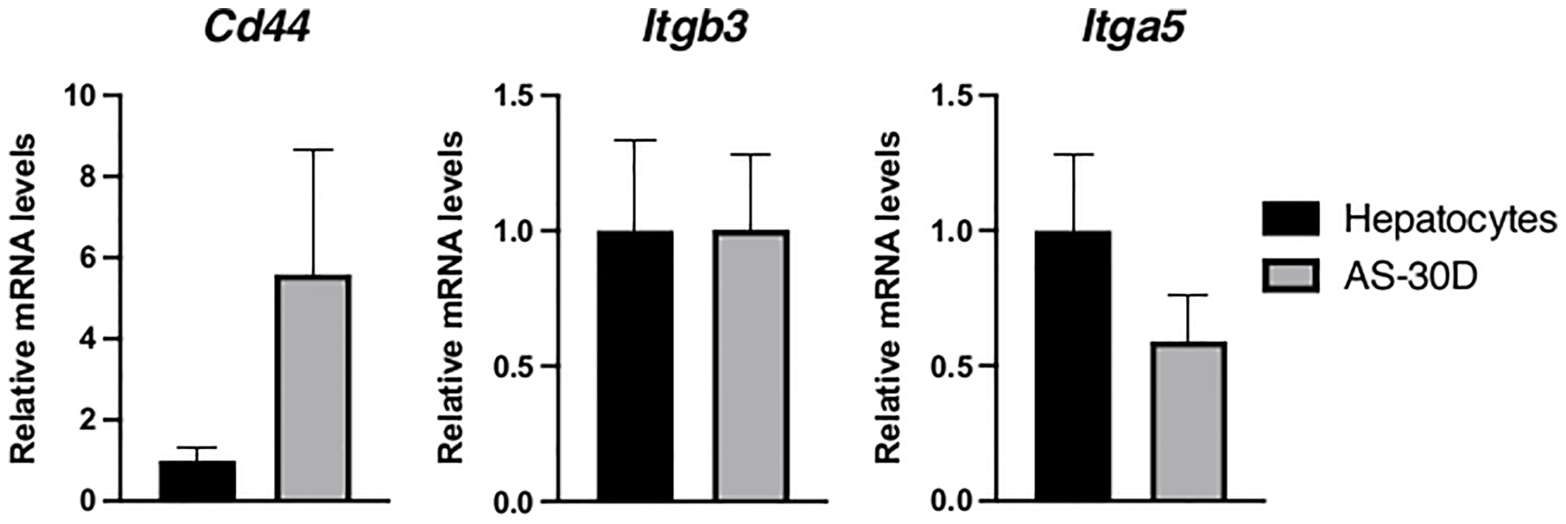
Gene expression of canonical receptors recognizing OPN in rat hepatocytes and AS-30D cells. Rat hepatocytes were freshly isolated, and AS-30D samples were obtained from cells from the ascitic fluid of experimental rats. Data represented as mean ± SD.

We analyzed *Cd44*, and several other members of the family of integrins, recognized to be the canonical receptors for OPN (10). Although *Cd44* showed the tendency to increase in AS-30D cells, the difference was not statistically significant. Regarding integrins, we analyzed those especially relevant in the context of cancer. *Itgb3* or integrin β3 mRNA, shows similar levels of expression between fresh hepatocytes and AS-30D cells, while *Itga5* or integrin αV mRNA, shows a lower expression in AS-30D cells (**Figure 5**).

### Plasma Membrane Ca^2+^-ATPase Transcripts

Taking into consideration that the control of cell calcium homeostasis has been known to be critical factor for the survival of most eukaryotic cells, we analyzed the expression of several PMCAs that contribute to maintain Ca^2+^ concentration at the nanomolar range. From these PMCA isoforms, a number of them have been recognized to play an important role not only in normal but also in neoplastic cells.

**Figure 6:**
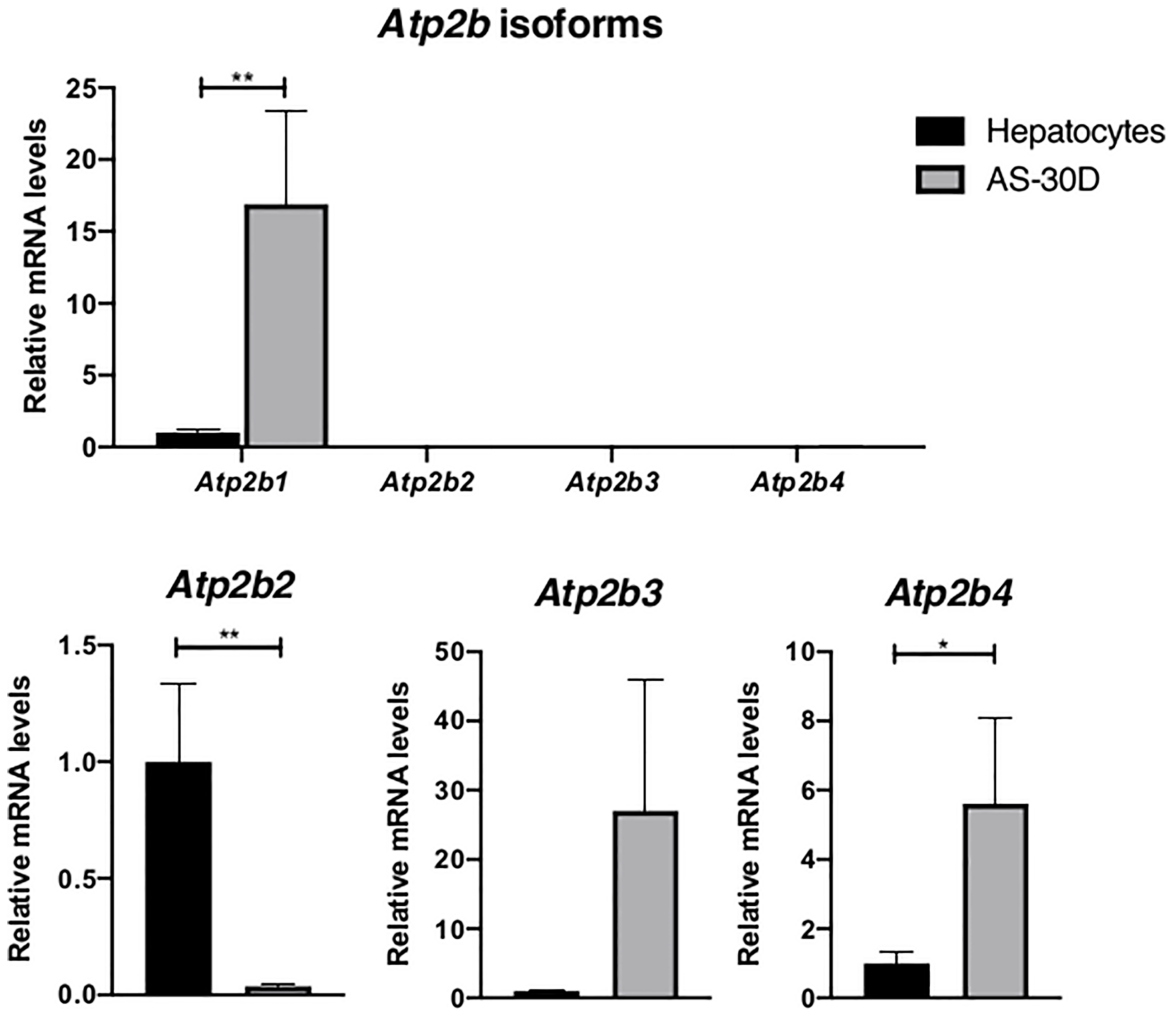
Expression of genes encoding for the plasma membrane Ca^2+^-ATPases (*Atp2b1-Atp2b4*) in rat hepatocytes and AS-30D cells. Rat hepatocytes were freshly isolated and AS-30D samples were obtained from cells from the ascitic fluid of experimental rats. Data represented as mean ± SD. *p-values ≤0.05; **p ≤0.01.

We observed that the housekeeping isoform *Atp2b1* (PMCA1) represents the isoform showing the highest level of expression with an important difference between AS-30D cells and normal hepatocytes (**Figure 6**). Although the expression of *Atp2b4*, also considered to be a housekeeping isoform, shows a level several orders of magnitude smaller, an increased level of this isoform is again found in tumor cells. Regarding the less abundant isoforms, *Atp2b2* showed a significant decrease in tumor cells, while *Atp2b3* showed an increasing but not significant trend in hepatoma cells.

### Protein Expression

**Figure 7:**
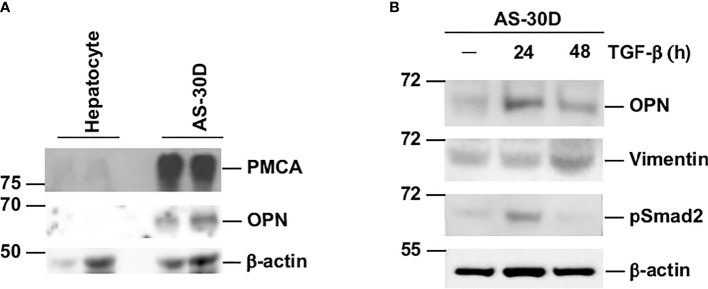
AS-30D cells express high protein levels of PMCA and OPN and increase the expression of mesenchymal markers after TGF-β stimulation. **(A)** Protein levels for PMCA and OPN in hepatocytes and AS-30D cells were detected by Western blot. One hundred micrograms of protein (first lane) and 200 μg (second lane) were run for each cell type. **(B)** AS-30D cells were treated with 2 ng/ml of TGF-β for 24 and 48 h OPN, vimentin, and phospho-Smad2 protein levels were detected by Western blot. One hundred micrograms of protein were run in each lane. β-Actin was used as a loading control.

Next, we proceeded to corroborate that the differences in mRNA concentration for some of the genes analyzed in normal hepatocytes and AS-30D cells were also reflected at the protein level. We measured by Western blotting the protein levels for PMCA1-4 and OPN. Our results show an elevated expression of PMCA and OPN in AS-30D cells compared with normal hepatocytes (**Figure 7A**). We expect this band corresponds to PMCA1, the isoform most abundant in liver. Similar results were obtained for OPN, showing higher protein levels detected in AS-30D cells compared with normal hepatocytes. These results enable us to corroborate that in the hepatoma AS-30D, there is a correlation between the high mRNA and protein levels detected for PMCA and OPN.

Finally, with the purpose to study if the AS-30D cells could be considered a suitable model *in vitro* to study EMT, we analyzed the effect of TGF-β, a potent inducer of EMT, upon the expression OPN and vimentin. AS-30D cells treated with TGF-β increased the expression of the mesenchymal markers OPN and vimentin, with the highest OPN expression at 24 h and vimentin at 48 h after TGF-β stimulation (**Figure 7B**). The activation of the TGF-β pathway was corroborated by the detection of phospho-Smad2 24 h after treatment.

## Discussion

Our results for the first time present a quantitative analysis of several genes encoding for cancer hallmarks employing the hepatocarcinoma transplantable model AS-30D. The use of this model and the analysis performed studying genes related to signaling molecules that mediate EMT, allowed us to propose that changes in the overall expression of these genes might be critical during cancer progression and important in the establishment of metastasis. AS-30D cells show high glycolytic activity and vascularization properties (3, 4), two essential hallmarks for HCC. Also, AS-30D cells are able to produce ascites, reported in HCC as a feature associated with bad prognosis, in rats 7 days after being inoculated. As in humans, the intraperitoneal accumulation of ascites in rats is related to the development of different etiologies where also kidney malfunction and sodium retention are involved (41). In a similar fashion, the presence of ascites in patients with HCC has been related to tumoral and cirrhosis factors, and a low survival rate (42).

Currently, there is not an animal model in use in the laboratory that exactly reproduces all the characteristics of HCC in humans. This is not surprising as it is cancer with a great degree of heterogeneity, in part due to the several etiologies that can cause it: chronic inflammation, viral infection, aflatoxin toxicity, alcohol, obesity, among others (43). However, our model of hepatoma AS-30D has similarities in the expression patterns for normal and cancer tissues for genes as FoxM1, Met, and especially Spp1 (**Supplementary Figure S1**). The gene profiles presented in this panel correspond to the dataset from Chen et al. (32), and other similarities can be found when using different datasets.

There are some advantages for using AS-30D cells over other models employed in the study of HCC. For example, the AS-30D model requires just a single inoculation to develop tumoral cells in the ascites after 1 week, making the procedure simple, cost-effective, and less time-consuming. These are all ideal characteristics for a model to be used in animals in high-throughput screenings for drug discovery.

Angiogenesis, the production of new blood vessels, allows the tumor to spread and to become metastatic. For this process to occur, permeability changes in the vascular endothelium mediated by proinflammatory cytokines, and growth factors such as VEGFα have to develop (2). In agreement with these observations, we found that cells collected from the ascites fluid overexpressed *Vegfα* when compared with hepatocytes, whereas *Angpt1* shows the opposite pattern. In lung tumors, it has been shown that angiopoietin-like factors also present this kind of behavior (44). Interestingly, an elevated expression of *Angpt1* in the normal hepatocyte, resembles the pattern observed during proliferation in livers undergoing the process of regeneration (45).

Linked to a proliferative, invasive, and metastatic phenotype in HCC (37, 45, 46), we further detected high levels of expression of factors essential for tumor progression such as *FoxM1*, *C-met*, and *Inppl1* in AS-30D cells compared with normal hepatocytes. While *FoxM1* and *C-met* are related to the active proliferative ability of cells in different types of cancer (46, 47), *Inppl1* is associated to a bad prognosis in HCC patients (38, 39). These data are consistent with the aggressiveness observed for the AS-30D model. On the other hand, in the context of HCC development, the epidermal growth factor receptor (EGFR) modulates relevant aspects of tumor progression such as proliferation, apoptosis, and metastasis (48). It has been shown to be overexpressed in HCC, and it is associated with a poor prognosis (49). In our model, we observed *Egfr* to present a low level of expression in comparison with normal hepatocytes. It has been reported that EGRF expression levels are heterogeneous in HCC, especially in tumor samples with metastatic features (50). In our case, this downregulation seems to allow HCC cells to undergo an EMT process and acquire a motile and migratory phenotype. Nevertheless, another possibility might be that we had detected a specific isoform of the molecule or even an EGFR mutant, both recognized as main contributors for the specific signaling pathway (51). Thus, it will be necessary in the near future to further investigate if the receptor also shows a low expression, and to explore in a closer detail, the activation state and modulatory mechanisms of this pathway with special emphasis on the crosstalk between EGFR and TGF-β. Also, since there are in the literature examples where transcripts levels do not correlate well with protein expression (9), we shall have to also study this scenario.

Our results suggest that TGF-β could play a central role during EMT in AS-30D cells *via* OPN. The expression of the mesenchymal markers OPN and vimentin induced by TGF-β, is a clear sign that AS-30D cells can undergo an EMT. The increase in OPN, will could lead to synergistic effect with the TGF-β in EMT, to potentiate the expression of mesenchymal markers. Accordingly, it has been reported that in epithelial cells, EMT is mainly driven by TGF-β signaling considered the most important inductor of the process (40), with the additional contribution of other signaling pathways such as Wnt/β-catenin, Sonic Hedgehog, integrins, and purinergic pathways (52–54). In the case of OPN, it has been proposed to induce EMT in HCC by upregulating the expression of Twist, a key regulator of EMT (55). Moreover, the participation of OPN as a stimulator of the glycolysis through binding to receptor αvβ3 and activation of the NF-kB signaling pathway has been recently reported in several human HCC cell lines (56). Although CD44 is one of the most important receptors for OPN signaling (57, 58), we only observe a marginal increase. It is likely that through an autocrine mechanism, osteopontin can stimulate cell migration. Another factor to keep in mind is that OPN interacts not only with CD44 or integrins but also *via* vimentin and MyD88, participating in all the stages of carcinogenesis through different signaling pathways (14). For many years, α-fetoprotein (AFP) has been used as the only marker for HCC diagnosis; however, there is not always a direct correlation between high levels of AFP and HCC (59). For that reason, additional markers such as cytokeratin19, des-γ-carboxythrombin or the Golgi protein 73, have been added in the diagnostic toolset for HCC (59). This set of markers includes also OPN, since *SPP1* is one of the most consistently overexpressed genes in HCC samples, and that matches our observations in AS-30D cells.

Although *CDH2*, which encodes for the protein N-cadherin, has been involved in EMT when studied in several cancers such as breast or pancreas cancer (60, 61), we did not find overexpression of the gene encoding this cell adhesion molecule in AS-30D cells. Previous reports have also presented controversial results where the presence of CDH2 has been found not only in normal liver tissue but also in the cytoplasm of metastatic hepatic cells (61). E- and N-cadherins when studied in liver samples from patients suffering HCC, show similar expression patterns where the loss of N-cadherin correlated with loss of E-cadherin (62). Nevertheless, work exploring by immunohistochemistry the expression of E- and N-cadherins, shows a higher expression of N-cadherin in normal liver than in HCC associated to hepatitis C virus (63).

To our surprise, we found that *Mmp2* with metalloprotease-2 as the end product, and *Acta2* encoding for α2-smooth muscle actin, are also underexpressed in tumor cells. There is the possibility that *Mmp2* and MMP2 are not expressed in AS-30D cells. Interestingly, Mizutani et al. suggested that mesothelial cells lining the peritoneum can promote the invasion of cancer cells, given that MMP2 is expressed mainly by the stromal fibroblasts adjacent to some tumors (64). Regarding *Acta2* expression, several papers report that in HCC lines exposed to TGF-β and primary hepatocytes isolated from patients who underwent hepatic resection, *Acta2* is not expressed (65). Moreover, they found that instead, γSMA could be considered a better marker and predictor for HCC progression. In addition, Morén et al. also showed that fibroblasts related to HCC express high levels of *ACTA2*/αSMA, while hepatocarcinoma cells (except HepG2 and SNU398 cells) do not (66).

For years now, it has been reported that calcium homeostasis is disturbed in cancerous process. PMCAs are part of the battery of proteins present in eukaryotic cells to deal with this kind of disturbance. We observed that *Atp2b1*, the most abundant transcript in normal cells, was highly overexpressed in AS-30D cells, supporting our previous studies (27). These higher levels of mRNA for *Atp2b1* in AS-30D correlated with the elevated protein levels of PMCA detected in AS-30D cells compared with fresh hepatocytes.

Different types of cancer cells present a particular expression profile for the PMCA isoforms. For example, *Atp2b1* (67) and *Atp2b2* are overexpressed in breast cancer cells (68), whereas in colon cancer, *Atp2b4/*PMCA4 were found to be upregulated (69) or with no significant changes (70). This overexpression of *Atp2b1* in the AS-30D cells, resembles changes observed in isolated hippocampal CA1 neurons showing epileptiform activity where it is known that calcium homeostasis is also altered (29). In both cases, it seems PMCA1 takes a preponderant role as a housekeeping isoform in the process to maintain or return to a normal cytoplasmic calcium level, independently if cells have acquired a neoplastic genotype. The overexpression of the transcript and the protein itself, might be considered an important survival mechanism since PMCA1 presents a lower Ca^2+^ affinity than other isoforms, as previously reviewed by us (71).

Given that HCC is one of the most frequent and aggressive types of cancer, it is of paramount importance to understand the processes that control EMT and reverse process, mesenchymal-epithelial transition (MET). The balance reached between them, most probably defines the progression of a liver injury towards a neoplastic process. In accordance with this possibility, it has been observed EMT in HCC and liver fibrosis where cells can express concomitantly, both epithelial and mesenchymal markers (72). Our results, that support this duality, suggesting that the hepatocarcinoma AS-30D is a suitable model to study relevant molecules of processes such as EMT. This has been corroborated by the detection of critical players in this process at the level of mRNA such as *Tgfb1* and *Spp1*, which encode for TGF-β and OPN, respectively, two key positive regulators of EMT (73). Most importantly, we corroborated that TGF-β induced an EMT in AS-30D cells, as it was shown by the increasing levels of the proteins OPN and vimentin.

The current concept to understand EMT in the context of cancer, refers rather to the presence of a population of cells showing a mixed phenotype where some cells are more epithelial-like while others show a more mesenchymal phenotype, instead of a “complete transition” between these two stages. This concept is based on the observation that many metastatic carcinomas show epithelial phenotypes and the difficulty to detect cells having undergone EMT in cancerous tissue *in vivo* (74). It is likely that the hepatoma AS-30D displays a hybrid phenotype as it is frequently observed in aggressive carcinomas in different tissues.

This study has allowed us to corroborate the overexpression of *Spp1* transcripts in the hepatoma AS-30D, as observed in human HCC samples. Furthermore, we observed a direct correlation between high mRNA levels and an elevated expression of the protein PMCA and OPN in AS-30D cells compared with hepatocytes. We also showed that TGF-β directly upregulates OPN and vimentin expression in AS-30D cells, an indication that this cell type is experiencing an EMT. Altogether, these findings suggest that the hepatoma AS-30D could be used to study EMT in HCC. We are certain that the study of EMT or the reverse process MET, using the transplantable hepatocarcinoma AS-30D model in rat, an immunocompetent animal could be a valuable tool in HCC research.

## Data Availability Statement

The raw data supporting the conclusions of this article will be made available by the authors, without undue reservation.

## Ethics Statement

The animal study was reviewed and approved by Animal Care and Use Committee Instituto de Fisiologia Celular Protocol JMO119(89)-17.

## Author Contributions

Conceived and performed the experiments: MB-O, BD-C, RG-V, and MS-G. Contributed to hepatocytes preparation: MS-G and MM-S. Contributed to the interpretation of results: MB-O, BD-C, RG-V, MS-G, MM-S, and JM-O. Original draft preparation: MB-O, BD-C, and JM-O. Supervised the project: MB-O, BD-C, and JM-O. All authors contributed to the article and approved the submitted version.

## Funding

MB-O was supported by the Wellcome Trust grant (104771/B/14/Z). This work was also supported by a grant from CONACYT (255778) awarded to JM-O.

## Conflict of Interest

The authors declare that the research was conducted in the absence of any commercial or financial relationships that could be construed as a potential conflict of interest.

The reviewer AZH declared a shared affiliation, with several of the authors, BDC, RGV, MSG, MMS, JMO, to the handling editor at the time of the review.

## Publisher’s Note

All claims expressed in this article are solely those of the authors and do not necessarily represent those of their affiliated organizations, or those of the publisher, the editors and the reviewers. Any product that may be evaluated in this article, or claim that may be made by its manufacturer, is not guaranteed or endorsed by the publisher.

## Supplementary Material

Supplementary Figure S1 Gene expression for *VEGFA*, *ANGPT1*, *FOXM1*, *MET*, *INPPL1*, *EGRF*, *TGFB1*, *MMP2*, *CDH2*, *ACTA2*, *CD44*, *ITGA3*, *ITGA5*, *ATP2B1*, *ATP2B2*, *ATP2B3*, and *ATP2B4* in normal liver and HCC samples. For all the genes, the data show was from Chen et al. (32), except for *ITGA5*, which is not included in this dataset; *ITGA5* data are derived from Mas et al. (31).
