# Supplementary material for: Quantitative Expression of Key Cancer Markers in the AS-30D Hepatocarcinoma Model
Source: Front Oncol. 2021 Oct 19;11:670292. doi: 10.3389/fonc.2021.670292 (PMC8561839; doi:10.3389/fonc.2021.670292)
Supplement: Supplementary Figure S1 — Gene expression for VEGFA, ANGPT1, FOXM1, MET, INPPL1, EGRF, TGFB1, MMP2, CDH2, ACTA2, CD44, ITGA3, ITGA5, ATP2B1, ATP2B2, ATP2B3, and ATP2B4 in normal liver and HCC samples. For all the genes, the data show was from Chen et al. (32), except for ITGA5, which is not included in this dataset; ITGA5 data are derived from Mas et al. (31). [file Image_1.pdf]

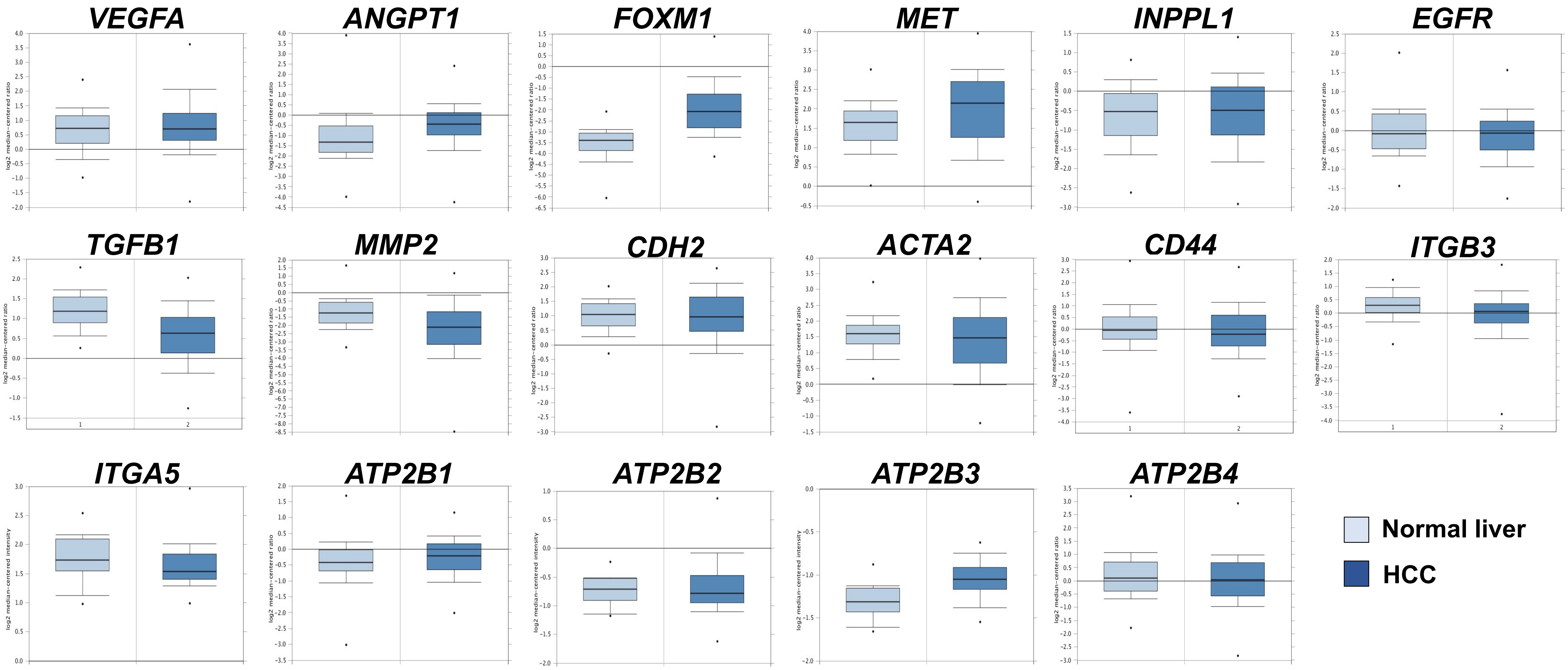
VEGFA
ANGPT1
FOXM1
MET
INPPL1
EGFR
TGFB1
MMP2
CDH2
ACTA2
CD44
ITGB3
ITGA5
ATP2B1
ATP2B2
ATP2B3
ATP2B4
Normal liver
HCC
